# Global research hotspots and trends in microglia in ischemic stroke

**DOI:** 10.3389/fimmu.2025.1622499

**Published:** 2025-10-31

**Authors:** Yingquan Liu, Yu Ye, Fan Dai, Lin Bai, Hongjie Ji, Xingxing Su, Peijia Hu, Hongliang Cheng

**Affiliations:** 1 The First Clinical College of Anhui University of Chinese Medicine, HeFei, China; 2 Graduate School Anhui University of Chinese Medicine, Hefei, China; 3 The Second Affiliated Hospital of Anhui University of Chinese Medicine, HeFei, China

**Keywords:** microglia, ischemic stroke, neuroinflammation, bibliometric analysis, visual analysis

## Abstract

**Objective:**

This study explores potential therapeutic strategies by analyzing the bibliometric analysis of microglia in ischemic stroke (IS) to identify the current status, hotspots, and trends in research.

**Methods:**

In this study, we visualized publications on IS and microglia indexed in the Web of Science Core Collection between January 1, 2010, and March 15, 2025, using VOSviewer and CiteSpace software. In addition, we optimized several visualization maps using Pajek and Scimago Graphica to present the analysis results more clearly and intuitively.

**Results:**

A total of 2,117 articles and reviews from 465 journals were included in the analysis. The number of publications reveals a steady increase over the years. China and the United States lead the field in terms of influence. Jun Chen from the University of Pittsburgh is the most influential scholar, and the *Journal of Neuroinflammation* is the most frequently cited journal among researchers. Through visual analysis of subject categories, keywords, and references, we found neuroinflammation to be the central mechanism in this research area. Therapeutic approaches primarily focus on using mesenchymal stem cells and extracellular vesicles, representing one of the most promising translational strategies for treating IS. Furthermore, innovative applications of neuroimaging technology and nanotechnology are facilitating the translation of basic research into clinical practice.

**Conclusion:**

This study uses bibliometric methods to summarize key findings in microglia-related IS research. The insights gained will provide valuable guidance and reference for developing new immunotherapeutic strategies based on microglia for more effective prevention and treatment of IS.

## 1 Introduction

Ischemic stroke (IS), characterized by the sudden occlusion of cerebral blood flow leading to focal neurological deficits, is a leading cause of death and long-term disability globally (1). It is estimated that approximately 7.6 million new cases occur globally each year, with 80% resulting from thromboembolic events caused by atherosclerosis or cardiogenic emboli (2). Although tissue-type plasminogen activator (tPA) intravenous thrombolysis and endovascular thrombectomy have revolutionized acute-phase treatment, their efficacy is highly time-dependent on the duration of treatment. Due to the narrow therapeutic window (< 4.5 h for tPA and < 24 h for thrombectomy), fewer than 10% of patients receive reperfusion therapy in a timely manner (3–5). Moreover, reperfusion can lead to secondary injuries, including oxidative stress, excitotoxicity, blood-brain barrier (BBB) disruption, and neuroinflammation, all of which can diminish the benefits of revascularization (6). Despite decades of research on neuroprotective drugs targeting excitotoxicity and calcium overload, these pharmacologic interventions have not translated into clinical success (7, 8). This demonstrates the limitation in our current understanding of IS pathomechanisms and underscores the urgent need to explore more effective treatment strategies.

As resident immune cells of the central nervous system (CNS), microglia are derived from yolk sac medullary progenitor cells and comprise 10%–15% of all glial cells (9, 10). These active “sentinels” continuously monitor the status of parenchymal cells through highly branched protrusions and constitute the brain’s first line of defense (11). Within minutes of the onset of cerebral ischemic injury, microglia are rapidly activated, initiating an inflammatory response in the brain (10, 12). Growing evidence suggests that immunomodulatory molecules (cytokines, chemokines, and trophic factors) released by activated microglia are a major driver of immune response following cerebral ischemia (13). These immune molecules are involved not only in the pathological processes that contribute to neurological damage during IS pathology (exacerbating neuroinflammation, disrupting the BBB, and triggering hemorrhagic transformation [HT]) but also in mechanisms of neurological restoration processes (suppressing neuroinflammation, reducing cerebral edema, and protecting white matter integrity) (13). Furthermore, molecules produced by other cell types, including endothelial cells, neuronal cells, and blood-derived immune cells, interact with microglia in a complex manner in the CNS, collectively influencing outcomes after IS (14–17). Given the intricate and multifaceted nature of microglial responses in IS, current studies targeting microglia and their associated molecular pathways have produced inconclusive and sometimes controversial results. Therefore, in-depth studies of microglia changes and their functions are necessary to develop new therapies for immunization in patients with IS.

Bibliometrics is a statistical methodology that quantitatively assesses the scholarly literature in a specific field to map the intuitive accumulation of knowledge, research trends, and collaborative networks (18). Recently, bibliometrics has been applied to IS and molecular cellular immunology, yielding valuable insights (19, 20). Despite the central role of microglia in mediating immune response within the ischemic brain microenvironment, no bibliometric analysis to date has specifically explored the role of microglia in IS. Consequently, this study utilizes bibliometric methods to examine the current landscape and key research themes at the intersection of microglia and IS, as well as the emerging trends of microglia in IS by analyzing the literature related to microglia in IS. This is crucial for a comprehensive understanding of IS pathophysiology and for finding new strategies to prevent or treat IS in the future.

## 2 Methods and materials

This study did not involve animal experiments or human testing; therefore, ethical review was not required.

### 2.1 Data collection

#### 2.1.1 Data retrieval and screening

On March 15, 2025, we searched for relevant publications on microglia in IS using the Web of Science Database Core Collection (WoSCC). The period was from January 1, 2010, to March 15, 2025. The search formulas used were as follows: TS = (“microglia” OR “microglias” OR “microglial cell” OR “cell, microglial” OR “microglial cells”) and TS = (“ischemic stroke” OR “ischemic strokes” OR “stroke, ischemic” OR “acute ischemic stroke” OR “acute ischemic strokes” OR “cryptogenic ischemic stroke” OR “cryptogenic stroke” OR “cryptogenic embolism stroke”). Initially, a total of 2,491 documents were retrieved. After filtering for English-language publications and excluding retracted papers, meeting abstracts, early access materials, and other ineligible document types, 2,352 records remained. Subsequent manual screening based on titles and abstracts was performed to further refine the selection. Studies were excluded according to the following criteria: ①Disease mismatch: studies not primarily focused on ischemic stroke (e.g., those focusing on cerebral hemorrhage, Alzheimer’s disease, or multiple sclerosis); ②Cellular focus mismatch: research not centered on microglia (e.g., studies mainly involving astrocytes, neurons, or neutrophils); ③Insufficient relevance: papers mentioning ischemic stroke and microglia only peripherally, without an in-depth investigation into their mechanisms or roles. Finally, the Full Record and Cited References of these 2,117 documents were exported in text format to prepare for the bibliometric analysis.

#### 2.1.2 Basis and validation for data source selection

This study selected the WoSCC as the sole data source, based on several key considerations. Firstly, WoSCC employs a rigorous journal selection mechanism and extensively covers high-impact academic journals, providing structured, reliable, and standardized data. This characteristic is crucial for ensuring the accuracy of bibliometric analysis and making the research findings comparable and reproducible (21). Secondly, the core objective of this study is to capture the mainstream research trends and core knowledge structures in the field of ischemic stroke and microglia, rather than aiming for comprehensive coverage without any omissions. The systematic coverage of core journals in this field by WoSCC aligns perfectly with this goal. Furthermore, WoSCC offers highly standardized and clean metadata (such as author keywords, references, etc.), which ensures optimal compatibility with mainstream bibliometric tools like VOSviewer and CiteSpace. This compatibility supports effective co-occurrence analysis, co-citation analysis, and other research methods that require high data consistency (21–23).

Although Scopus is renowned for its extensive coverage, the database includes a significant number of documents that have not undergone strict peer review, such as preprints and clinical case reports. This leads to notable heterogeneity in data standardization, completeness, and overall academic quality (24). Similar issues are observed with the PubMed database (25). Given these characteristics, Scopus and PubMed may not be the preferred choices for bibliometric studies that require high data quality and consistency. Moreover, combining databases from multiple sources could introduce significant confounding factors due to inconsistencies in data formats, indexing rules, and inclusion criteria. This not only increases the complexity of data cleaning but also raises the risk of subjective bias, potentially compromising the reliability of the final core conclusions (23). Therefore, such databases are more suitable as supplementary analysis tools when the coverage of WoSCC is limited and there is a substantial disparity in the number of documents available in Scopus and PubMed. They can be used to complement and validate the primary research findings.

To evaluate and validate the representativeness of WoSCC within the scope of this study, we conducted a preliminary search using the same retrieval strategy in Scopus. After applying uniform screening criteria, Scopus yielded 2,238 relevant documents, compared to the 2,117 documents retrieved from WoSCC. This result shows no significant increase in the number of documents (see **Supplementary Material 1** for details). This outcome indirectly indicates that WoSCC has effectively covered the majority of core literature in the field of ischemic stroke and microglia that meets the high standards required for bibliometric analysis. It demonstrates good representativeness and can reliably reflect the mainstream research trends and knowledge structures within this domain.

### 2.2 Data standardization

This study standardized data for country or area names and keywords (26). For country or region names: (i) Country name abbreviations are replaced with internationally recognized names, such as “United States” is replaced with “USA;” (ii) The name of the region should be harmonized with the name of the country to which it belongs, such as “Scotland” and “England” should be harmonized as “United Kingdom.” Keywords: (i) Artificial merging of synonyms, such as “brain ischemia” and “cerebral-ischemia,” should be merged into “cerebral ischemia;” (ii) unification of plural forms into singular forms, such as “astrocytes” into “astrocyte”.

### 2.3 Selection and application strategies for visualization tools

To comprehensively and multi-dimensionally reveal the knowledge structure of the research field, this study integrated two mainstream bibliometric software tools, VOSviewer (version 1.6.19) and CiteSpace (advanced version 6.4.R1), for network construction and visualization analysis. Each tool was applied to specific analytical tasks based on its core functional strengths to ensure the scientific rigor of the methods and the validity of the results.

Firstly, we leveraged CiteSpace’s powerful data preprocessing capabilities to organize and output the data from the 2,117 relevant documents selected through our screening process. These processed data were then imported into the software for in-depth analysis. VOSviewer excels in network layout and cluster display, and its integration with visual optimization software such as Pajek and Scimago Graphica facilitates the presentation of clear and robust large-scale network structures. In these networks, node sizes represent activity intensity, and line thickness indicates the strength of collaboration. Therefore, we prioritized the use of VOSviewer to generate visualization maps for collaborations among countries/regions, various institutions, authors, citation relationships of journals, and keyword co-occurrence.

**Figure 1:**
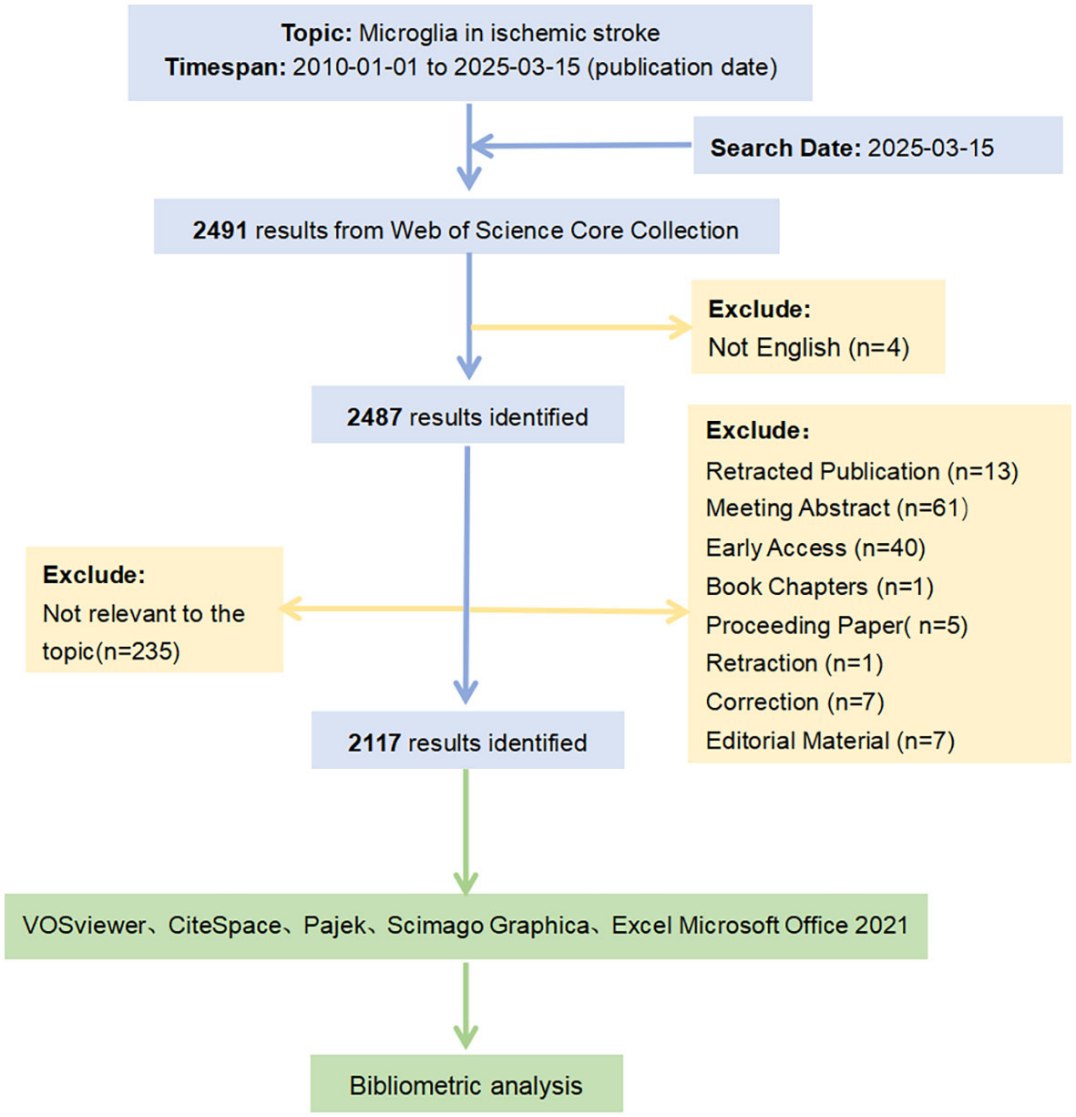
Flowchart for publication identification and analysis.

A key advantage of CiteSpace is integrating multiple independent networks into a comprehensive network, facilitating a systematic study of the relevant publications (27). Accordingly, we used this functional feature to generate clustered maps of subject categories and references. In addition, CiteSpace’s time-slicing technique was applied to construct a time series of evolving network models and generate timeline clustering maps for keywords. We also conducted a “burst test” on subject categories, keywords, and references using CiteSpace to identify emerging trends and bursts in specific research areas. Finally, Microsoft (Excel Office 2021) was used to manage, filter, and standardize the raw data downloaded from the WoSCC database and to create relevant tables illustrating the academic impact of the analyzed topics in the field. The flowchart detailing the paper screening and analysis process is depicted in **Figure 1**.

## 3 Results

### 3.1 Publication trends

**Figure 2:**
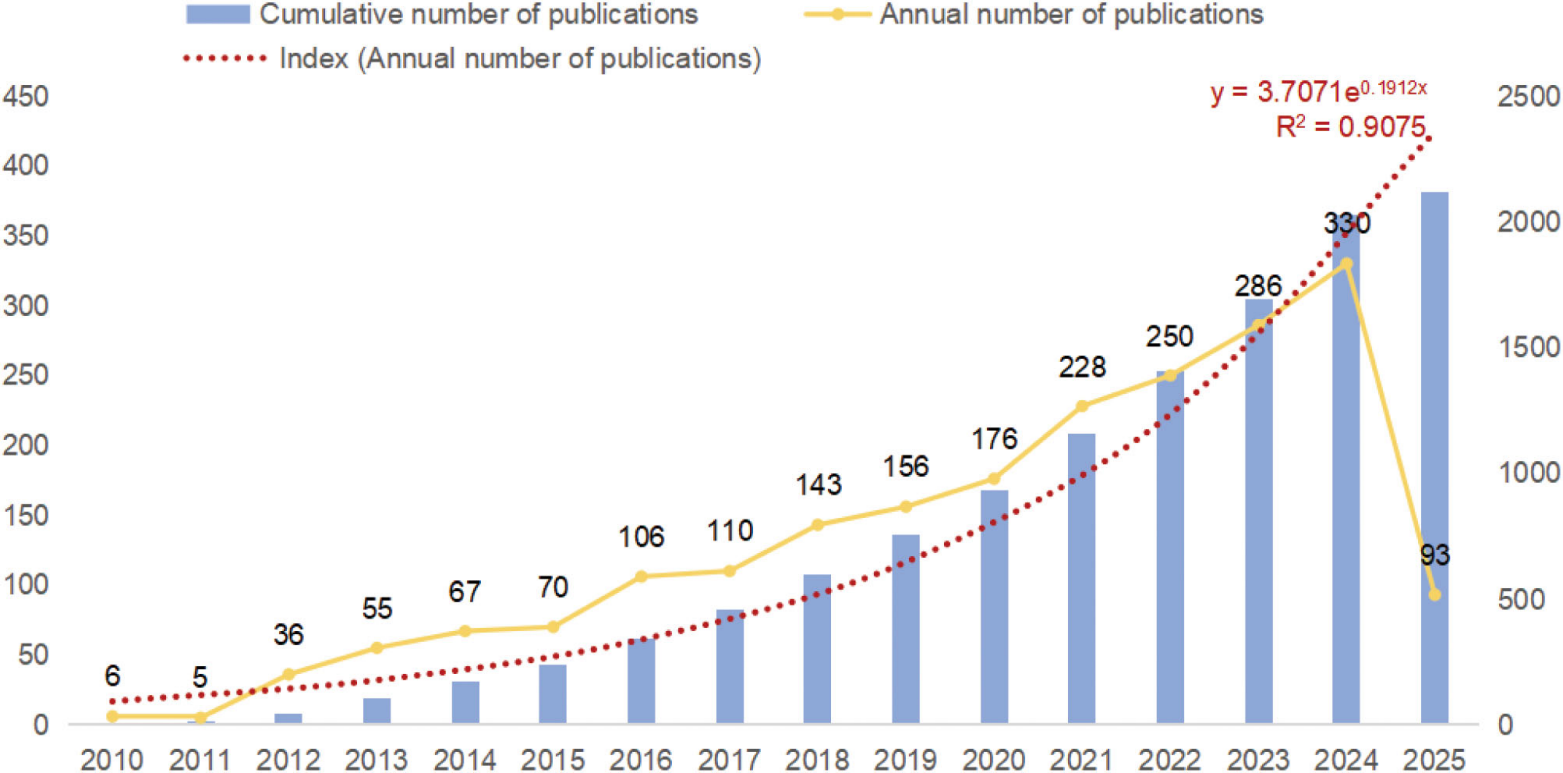
Trends in the number of publications.

The final selection of 2117 publications contained 1774 articles (83.80%) and 343 reviews (16.20%). **Figure 2** illustrates the trend of publications on microglia in IS. It can be observed that the growth in the number of relevant publications was relatively flat until 2015, but since 2015, researchers’ interest in microglia in IS has increased significantly, and the growth in the number of publications has accelerated significantly. The average annual publications rose from 33.8 ± 29.7 during 2010–2014 to 177.0 ± 81.7 from 2015 onward, with an independent samples t-test indicating a significant difference (*t* = 4.11, *P* = 0.001). In 2016, the number of publications in the field surpassed 100 for the first time; the growth is particularly significant in 2021, reaching 228, indicating that the role of microglia in IS has attracted widespread attention from scholars. Notably, 93 relevant publications have been published in just under one quarter between January 1 and March 15, 2025, and the number of publications for the whole year of 2025 is expected to exceed 400 articles. Overall, microglia remains a hotspot in IS research and bodes well in future research directions. Meanwhile, more studies on microglia in IS are expected to be published.

### 3.2 Analysis of countries and institutions

**Figure 3:**
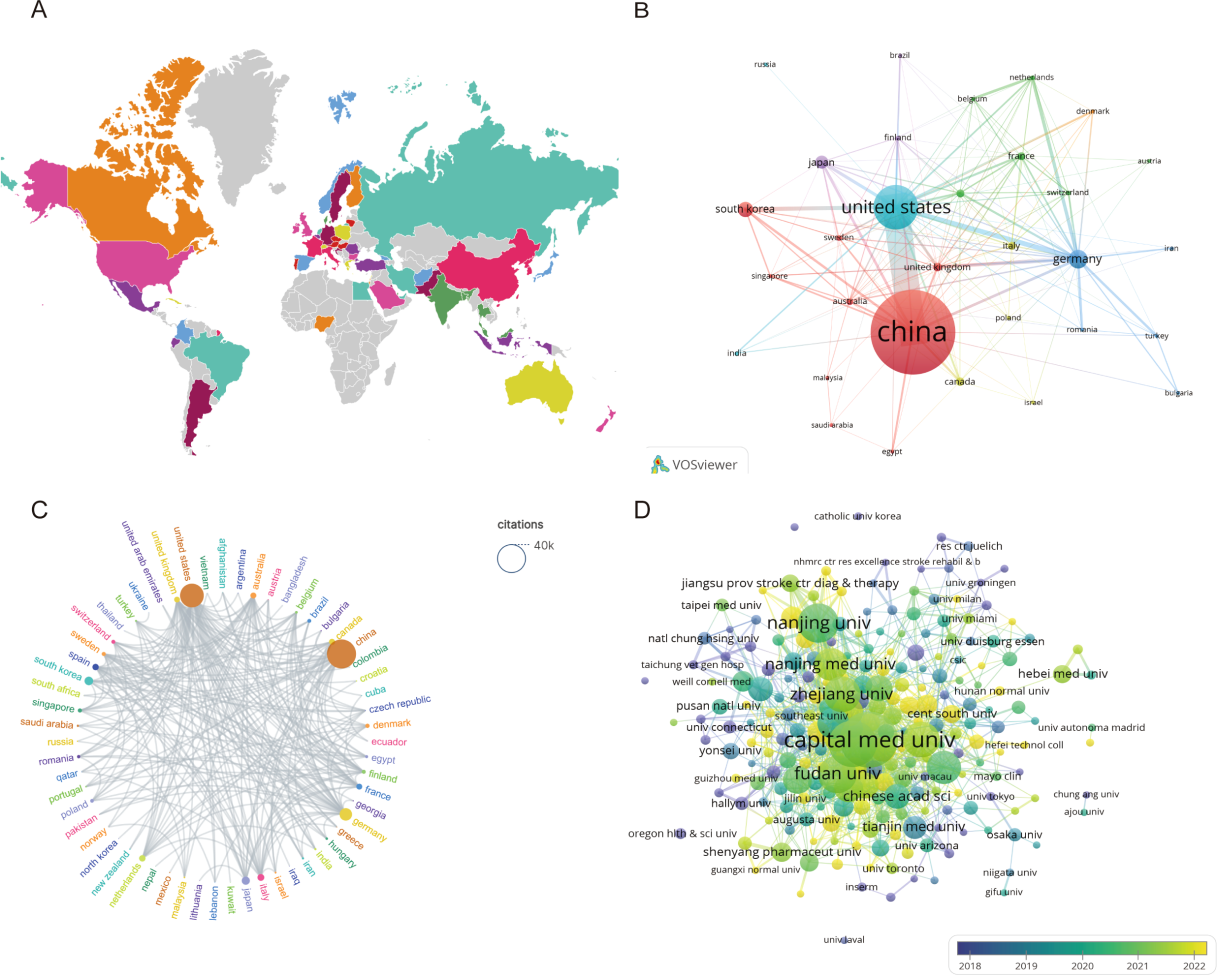
Visualization of countries and institutions involved in research on the microglia in IS. **(A)** Global distribution of microglia research results in IS. **(B)** Cooperation network of different countries in the field of microglia in IS. **(C)** National Citation Cooperation Network. **(D)** Collaborative network between research institutions in microglia in IS.

A total of 60 countries have published studies on IS microglia. **Table 1** lists the 10 most productive countries in this field, with China (1,240 papers), the United States (496 papers), and Germany (168 papers) making the most significant contributions. Additionally, the citation analysis in **Table 1** illustrates that both China and the United States have accumulated more than 20,000 citations, with 38,582 and 25,742 citations, respectively. However, the average number of citations per paper in the United States is almost twice that of China, at 51.90 and 31.11, respectively. This suggests that although China dominates in terms of the total number of papers and overall research output, the academic achievements of the United States are relatively more recognized internationally. As depicted in **Figure 3**, a visual map of the national and institutional collaboration networks was generated using VOSviewer. Moreover, country collaboration network data were imported into Scimago Graphica to create a world geographic distribution map of research findings and a country citation network view. **Figure 3A** visualizes the global geographic distribution of studies in this field. In the country citation network map indicated in **Figure 3B**, the citation relationships between countries with more than five published articles are presented. In this map, the node represents different countries, the node colors indicate different clusters, and the node size is proportional to the number of published articles. **Figure 3C** illustrates the country citation network, where China and the United States exhibit the most prominent influence. Overall, these countries have various collaborative relationships, with the strongest links observed between China, the US, and Germany. This suggests they are crucial in promoting international cooperation in this field.

**Table 1:** Top 10 most productive countries in the field of microglia in IS.

Rank	Countries	Publications	Citations	Average citations	Total link strength
1	China	1240	38582	31.11	207
2	United States	496	25742	51.90	252
3	Germany	148	7173	48.47	100
4	South Korea	110	3370	30.64	38
5	Japan	89	3022	33.96	44
6	United Kingdom	50	1504	30.08	54
7	Canada	47	1667	35.47	23
8	Italy	43	2148	49.95	27
9	Spain	43	1845	42.91	43
10	France	42	1602	38.14	36

According to the VOSviewer analysis, the above 2,117 papers were contributed by 1,993 different institutions, among which the top 10 institutions are listed in **Table 2**. It can be found that nine of the top 10 institutions are from China, contributing a total of 492 articles. The top three institutions in the citation impact analysis were Capital Medical University (3,749 citations), Fudan University (3,062 citations), and the University of Pittsburgh (3,041 citations). Notably, the University of Pittsburgh ranked first with an average citation count of 76.03, indicating a very high academic influence in the relevant fields. Strengthening cooperation with this institution may promote further development in this field. **Figure 3D** indicates the relatively dense collaborative network among the issuing institutions in microglia research in IS. Not surprisingly, China occupies a central position in the entire map of the cooperative network. Therefore, there is good reason to believe collaboration with Chinese institutions will greatly benefit research development in this field. Furthermore, it can be noticed from **Figure 3D** that the nodes in yellow and green have the strongest and widest distribution of connections, which suggests that the period between 2021 and 2022 and beyond is the most intensive period of inter-institutional collaboration.

**Table 2:** Top 10 most productive institutions in the field of microglia in IS.

Rank	Institutions	Publications	Citations	Average citations	Countries
1	Capital Medical University	92	3749	40.75	China
2	Shanghai Jiao Tong University	71	2728	38.42	China
3	Nanjing University	56	2089	37.30	China
4	Fudan University	52	3062	58.88	China
5	Sun Yat-sen University	49	1323	27.00	China
6	Zhejiang University	48	1715	35.73	China
7	Huazhong University of Science & Technology	44	2449	55.66	China
8	Nanjing Medical University	43	1201	27.93	China
9	University of Pittsburgh	40	3041	76.03	United States
10	China Pharmaceutical University	37	1326	35.84	China

### 3.3 Analysis of authors

**Figure 4:**
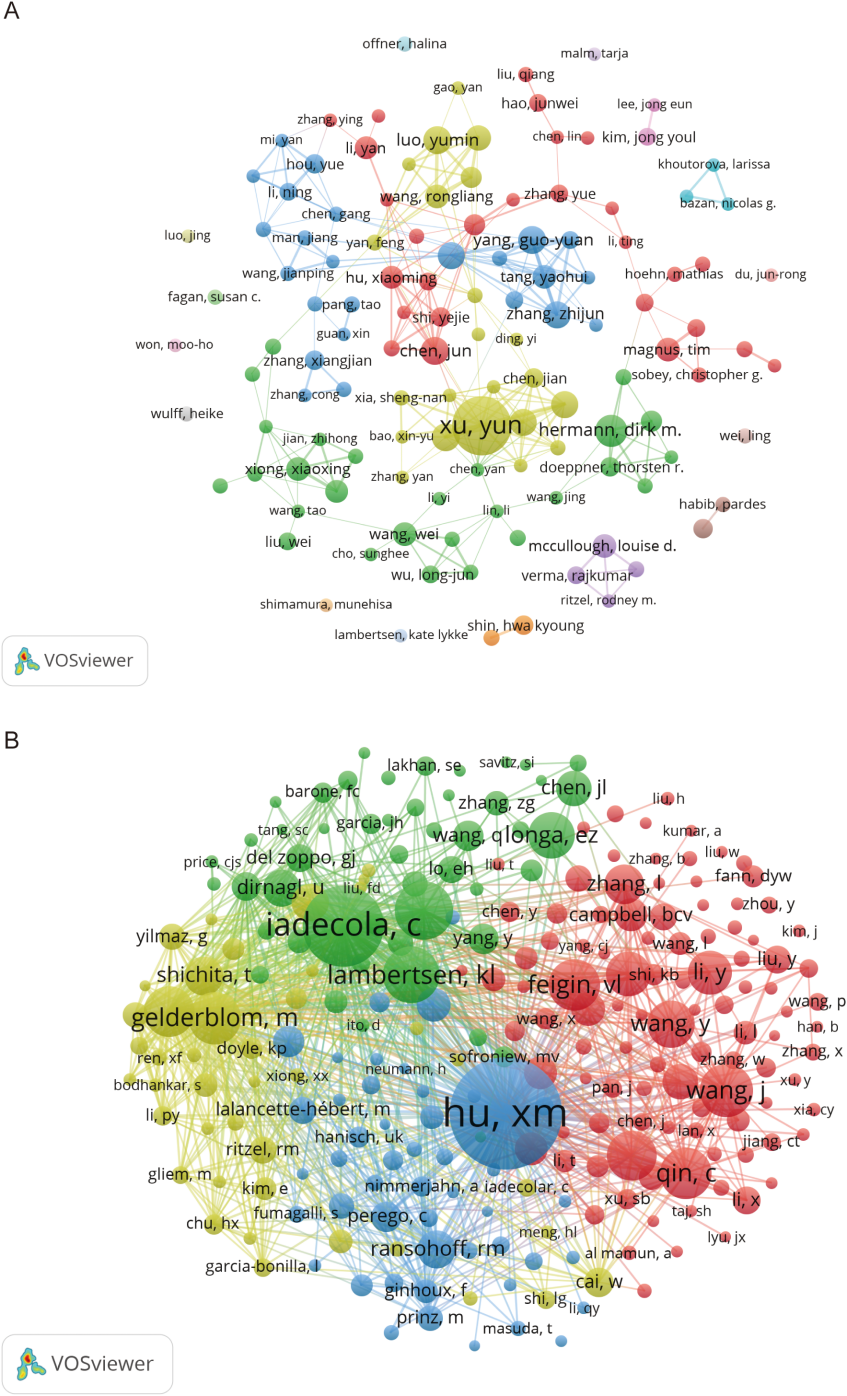
Visualized network mapping of relevant authors and co-cited authors in the field of microglia research in IS. **(A)** Visualization of authors that have published 7 or more papers. **(B)** Visualization of co-cited authors with at least 50 citations.

According to VOSviewer analysis, there are 11,753 authors in the field of microglia research in IS, and 300 authors have published at least five papers. **Table 3** lists the top 10 based on the number of papers published in this field of research, with the top three most prominent contributors being Yun Xu from Nanjing University (42 papers), Dirk M. Hermann from Ruhr University Bochum (20 papers), and Jun Chen from the University of Pittsburgh (17 papers). University of Pittsburgh (17 articles). According to the citation analysis in **Table 3**, Jun Chen ranks first in total and average citations, far surpassing other authors. This highlights his outstanding influence and authority in the field. His research primarily focuses on microglia activation and polarization (28), the role of microglia in mediating the neurorestorative effects of regulatory T cells (Tregs) (29), and strategies for achieving neuroprotection by targeting microglia to inhibit neuroinflammation (30). We used VOSviewer software to generate a collaborative network map of core and co-cited authors in microglia research in IS, as depicted in **Figure 4**. **Figure 4A** illustrates the cooperative relationships among 124 core authors, each of whom has published no fewer than seven publications. It can be observed that although several relatively stable collaborative groups have formed, there is a lack of close cooperation between these groups. This indicates an urgent need to strengthen collaboration and communication among the core authors, which would facilitate more rapid advancement of the field. **Figure 4B** indicates the clustering of co-cited authors in four different colors. There are 48,684 co-cited and 244 authors who have been cited at least 50 times or more. Among them, Xiaoming Hu from the University of Pittsburgh was the most cited author with 603 citations, and his research results primarily revealed the polarization phenomenon of microglia/macrophages following focal cerebral ischemic injury and their dual, opposing mechanisms of action (31).

**Table 3:** Top 10 most productive authors in the field of microglia in IS.

Rank	Authors	Publications	Citations	Average citations	Organizations
1	Yun Xu	41	1405	34.27	Nanjing University
2	Dirk M. Hermann	20	648	32.40	Ruhr University Bochum
	Jun Chen	17	2174	127.88	University of Pittsburgh
4	Xiang Cao	17	382	22.47	Nanjing University
5	Guo-Yuan Yang	17	1146	67.41	Shanghai Jiao Tong University
6	Yumin Luo	17	828	48.71	Capital Medical University
7	Zhijun Zhang	16	1054	65.88	Shanghai Jiao Tong University
8	Chang Liu	16	409	25.56	Shanghai Jiao Tong University
9	Xinyu Bao	16	172	10.75	Nanjing University
10	Haiping Zhao	15	782	52.13	Capital Medical University

### 3.4 Analysis of journals

**Figure 5:**
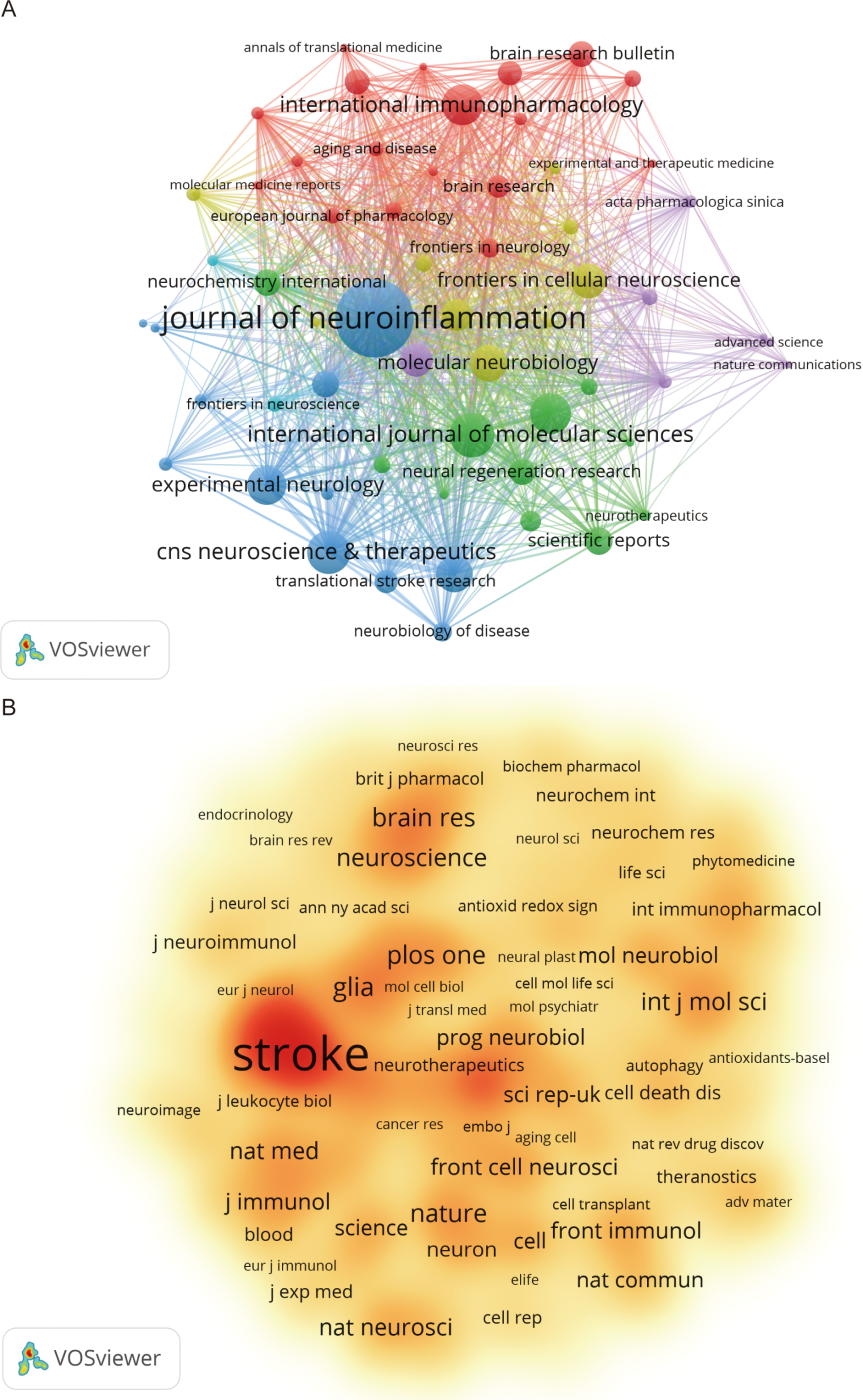
Visualized network mapping of relevant journals and co-cited journals in the field of microglia research in IS. **(A)** Visualization of journals that have published 5 or more papers. **(B)** Visualization of co-cited journals with at least 100 citations.

According to VOSviewer analysis, 465 journals published papers on microglia research in IS, of which 101 journals published more than five papers. **Figure 5A** indicates the citation visualization network of these 101 journals, displaying five major clusters in different colors. The top 10 journals by number of publications are listed in **Table 4**, collectively publishing 504 articles, which accounts for 23.81% of the total number of publications in the field. The *Journal of Neuroinflammation* (97 papers), *International Journal of Molecular Sciences* (53 papers), and *CNS Neuroscience & Therapeutics* (51 papers) contributed the most articles, with the top three journals all located within the Q1 division of the JCR. In the citation analysis (**Table 4**), the *Journal of Neuroinflammation* leads significantly with 5,490 citations and an impressive Impact Factor of 9.3, topping the list of the 10 most productive journals regarding publications and fully demonstrating its strong influence in the field. Besides, the *Journal of Cerebral Blood Flow and Metabolism* ranked first in terms of average citations per article, with an average list of 71.07 citations, demonstrating that the scientific results published in the journal are widely recognized and highly regarded in the field. Among the 5,386 cited journals analyzed, 237 were cited more than 100 times. **Table 5** lists the top-cited journals, with *Stroke* (7,330 citations), *Journal of Cerebral Blood Flow and Metabolism* (4,702 citations), and *Journal of Neuroinflammation* (3,700 citations) in the top three. **Figure 5B** indicates a visual density plot of journals with at least 100 citations, with darker colors representing higher citation counts.

**Table 4:** Top 10 most published journals in the field of microglia in IS.

Rank	Journals	Publications	Citations	Average citations	2023 JCR (IF)
1	Journal of Neuroinflammation	97	5490	56.60	Q1(9.3)
2	International Journal of Molecular Sciences	53	1864	35.17	Q1(4.9)
3	CNS Neuroscience & Therapeutics	51	1803	35.35	Q1(4.8)
4	International Immunopharmacology	48	1115	23.23	Q2(4.8)
5	Neuroscience	48	1073	22.35	Q2(2.9)
6	Experimental Neurology	45	1293	28.73	Q1(4.6)
7	Journal of Cerebral Blood Flow and Metabolism	42	2985	71.07	Q1(4.9)
8	Molecular Neurobiology	42	764	18.19	Q1(4.6)
9	Frontiers in Cellular Neuroscience	40	1778	44.45	Q2(4.2)
10	Frontiers in Immunology	38	1987	52.29	Q1(5.7)

**Table 5:** Top 10 most cited journals in the field of microglia in IS.

Rank	Journals	Citations	2023 JCR (IF)
1	Stroke	7330	Q1(7.8)
2	Journal of Cerebral Blood Flow and Metabolism	4702	Q1(4.9)
3	Journal of Neuroinflammation	3700	Q1(9.3)
4	Journal of Neuroscience	3315	Q1(4.4)
5	Proceedings of the National Academy of Sciences of the United States of America	2155	Q1(9.4)
6	Glia	1833	Q1(5.4)
7	Brain Research	1757	Q3(2.7)
8	PLOS ONE	1721	Q1(2.9)
9	Nature	1709	Q1(50.5)
10	Experimental Neurology	1610	Q1(4.6)

### 3.5 Analysis of subject categories

**Figure 6:**
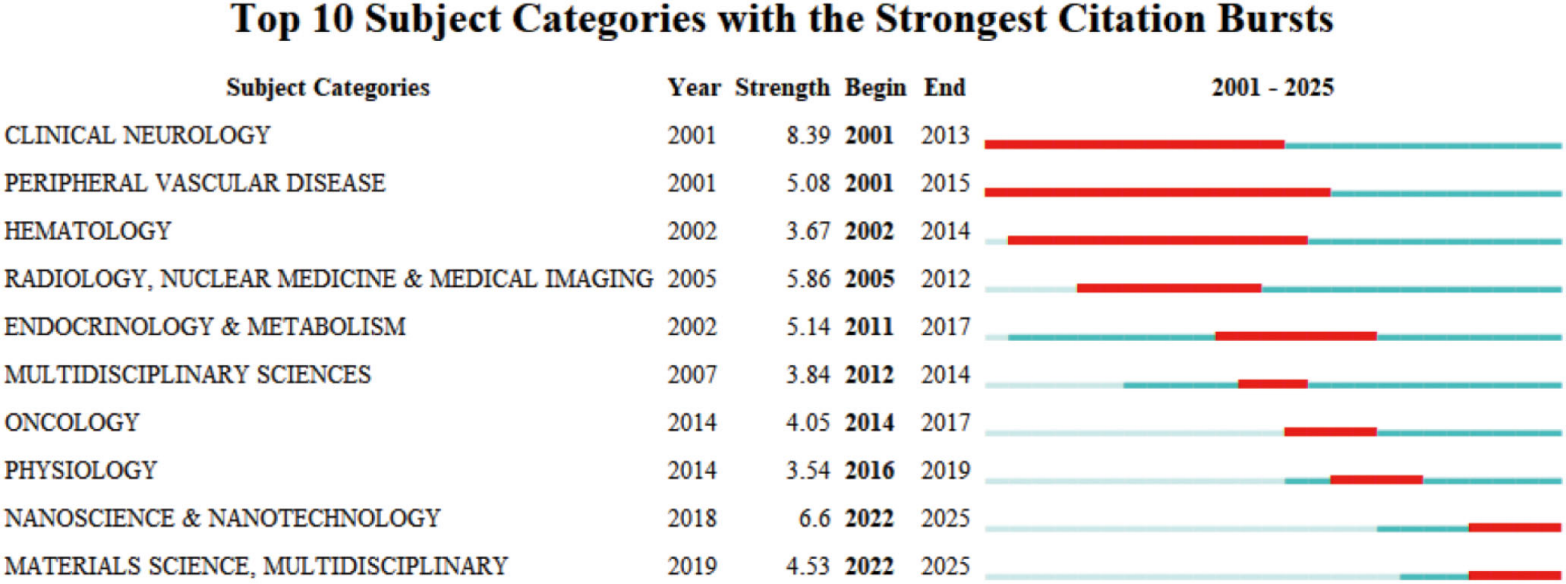
Top 10 subject categories for burst strength in IS microglia research.

The subject categories of 2,117 papers were burst-tested using CiteSpace to identify hotspots at the front of the microglia field in IS. The subject categories of 2,117 papers were mutated using CiteSpace to identify hotspots at the front of the microglia field in IS. **Figure 6** demonstrates the top 10 subject categories in terms of burst strength. The red timeline indicates the duration of the burst, while the burst strength quantifies the level of popularity or attention for each disciplinary category. The top three subjects in terms of burst strength are “CLINICAL NEUROLOGY,” “RADIOLOGY, NUCLEAR MEDICINE & MEDICAL IMAGING,” and “ENDOCRINOLOGY & METABOLISM.” This suggests that clinical manifestations, diagnosis, and treatment of neurological disorders, as well as systems for monitoring the dynamics of metabolic homeostasis of glial cells in the brain, need to depend on neuroimaging to provide evidence of structural and functional aspects. In addition, the strengths of the bursts that continue to this day are “NANOSCIENCE & NANOTECHNOLOGY,” “MATERIALS SCIENCE, MULTIDISCIPLINARY.” This suggests that the application of nanotechnology and the cross-fertilization of multidisciplinary fields will become the future trend in microglia research in IS.

### 3.6 Analysis of keywords

**Figure 7:**
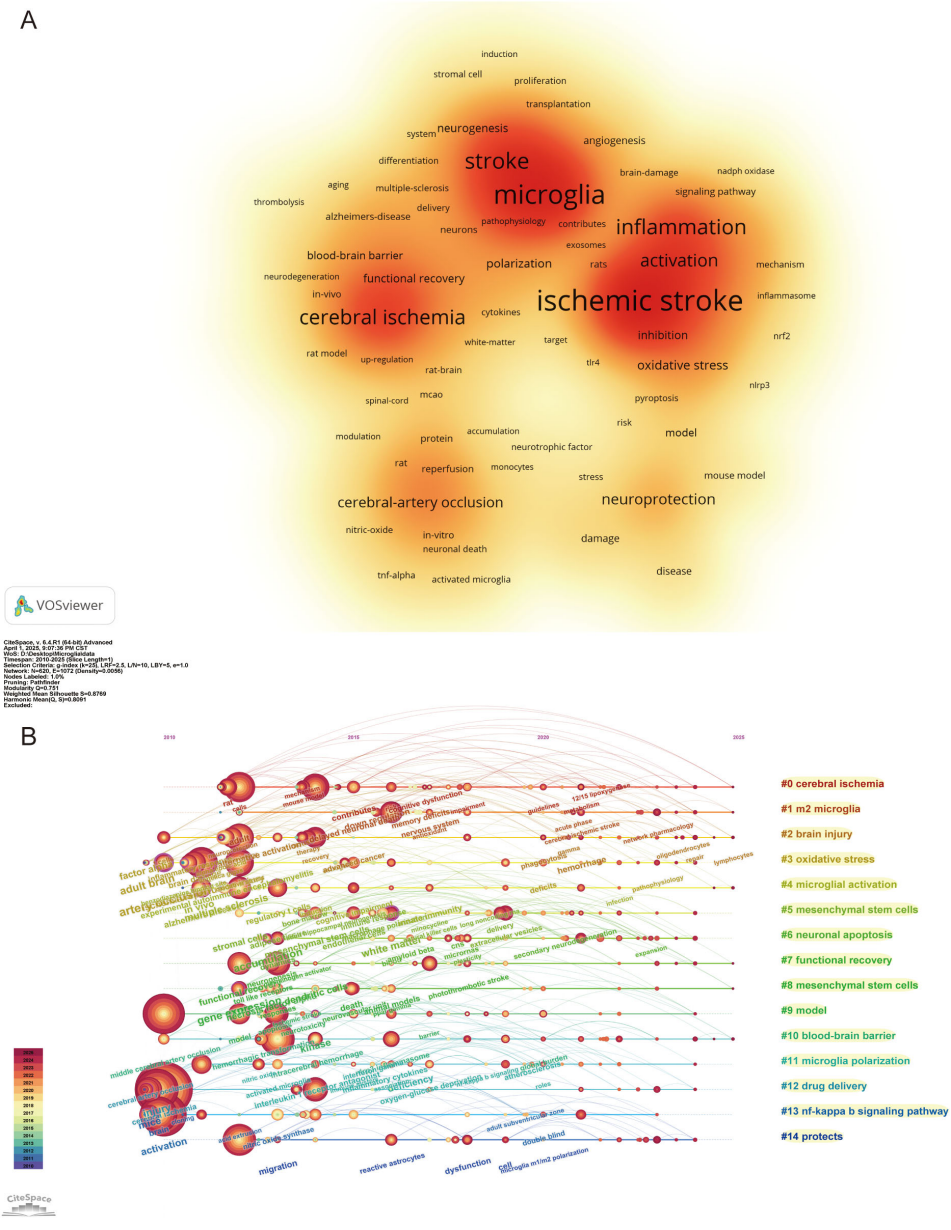
Visualization of keywords in the field of IS microglia research. **(A)** Visualization density mapping of 152 keywords with a frequency of at least 20 occurrences. **(B)** Timeline visualization of the first 15 keyword clusters.

Keywords in scientific papers often reveal the core theme of the research. By analyzing the frequency of keywords and their co-occurrence patterns, essential research directions and emerging trends in the field can be effectively identified. In this study, keywords were analyzed using VOSviewer software, and 6,710 keywords were identified, of which 152 keywords exhibit a frequency of no less than 20 occurrences. **Figure 7A** displays a density visualization map of these keywords, with five main clusters, where darker colors in each cluster indicate a higher frequency of the keyword. The primary keywords with high frequency in the map are “ischemic stroke,” “microglia,” “stroke,” “inflammation,” “cerebral ischemia,” “neuroinflammation,” “activation,” “injury,” “expression,” and “neuroprotection.” Together, these high-frequency keywords reveal that IS triggers cerebral ischemia, which activates microglia and leads to tissue damage and neuroprotection. Simultaneously, these keywords indicate that precise regulation of microglia function to realize the synergistic effect of inflammation inhibition and neuroprotection has become a hot spot in the current study.

**Figure 8:**
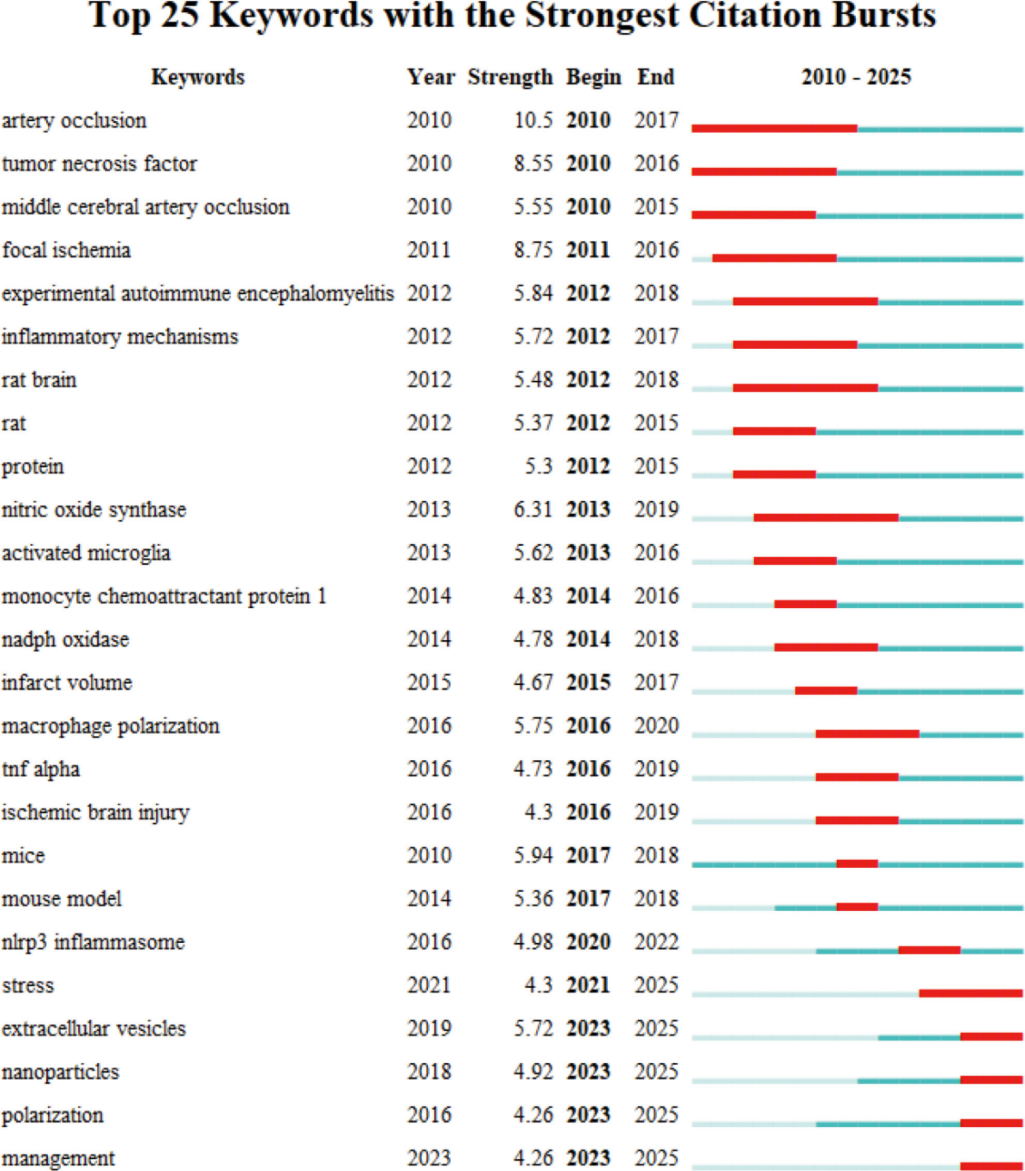
Top 25 keywords for burst strength in IS microglia research.

We used CiteSpace to cluster and analyze the keywords to further explore the research hotspots and trends in the field of IS microglia. The analysis produced 20 clusters, and **Figure 7B** depicts a timeline view of the top 15 keyword clusters. Cluster #0 cerebral ischemia, cluster #1 m2 microglia, cluster #2 brain injury, cluster #4 microglial activation, cluster #7 functional recovery, cluster #8 mesenchymal stem cells (MSCs), and cluster #10 BBB. These clusters are recent hot topics, primarily focusing on pathological mechanisms and therapeutic strategies. These themes not only reflect the focus of current research but also foreshadow future research directions. To further explore the research frontiers and evolutionary trends in this field, we used CiteSpace to conduct burst detection on keywords and generated a map of the 25 keywords with the highest burst strength (**Figure 8**). In the figure, red lines indicate the duration during which the corresponding topics were active research hotspots, while the burst strength values quantify the peak levels of attention these keywords received within specific time frames. The analysis revealed that the most prominent burst keywords in recent years include: “stress” (2021–2025), “extracellular vesicles” (2023–2025), “nanoparticles” (2023–2025), “polarization” (2023–2025), and “management” (2023–2025). These continuously emerging research topics indicate that the field is expanding in both the exploration of micro-mechanisms and clinical translational applications. Specifically, this includes foundational studies on microglial polarization and stress mechanisms, as well as innovative therapeutic strategies based on extracellular vesicles and nanotechnology. These cutting-edge directions not only reflect the increasing academic focus on neuroimmune regulation and precision intervention but also provide valuable references for predicting future research foci.

### 3.7 Analysis of references

**Figure 9:**
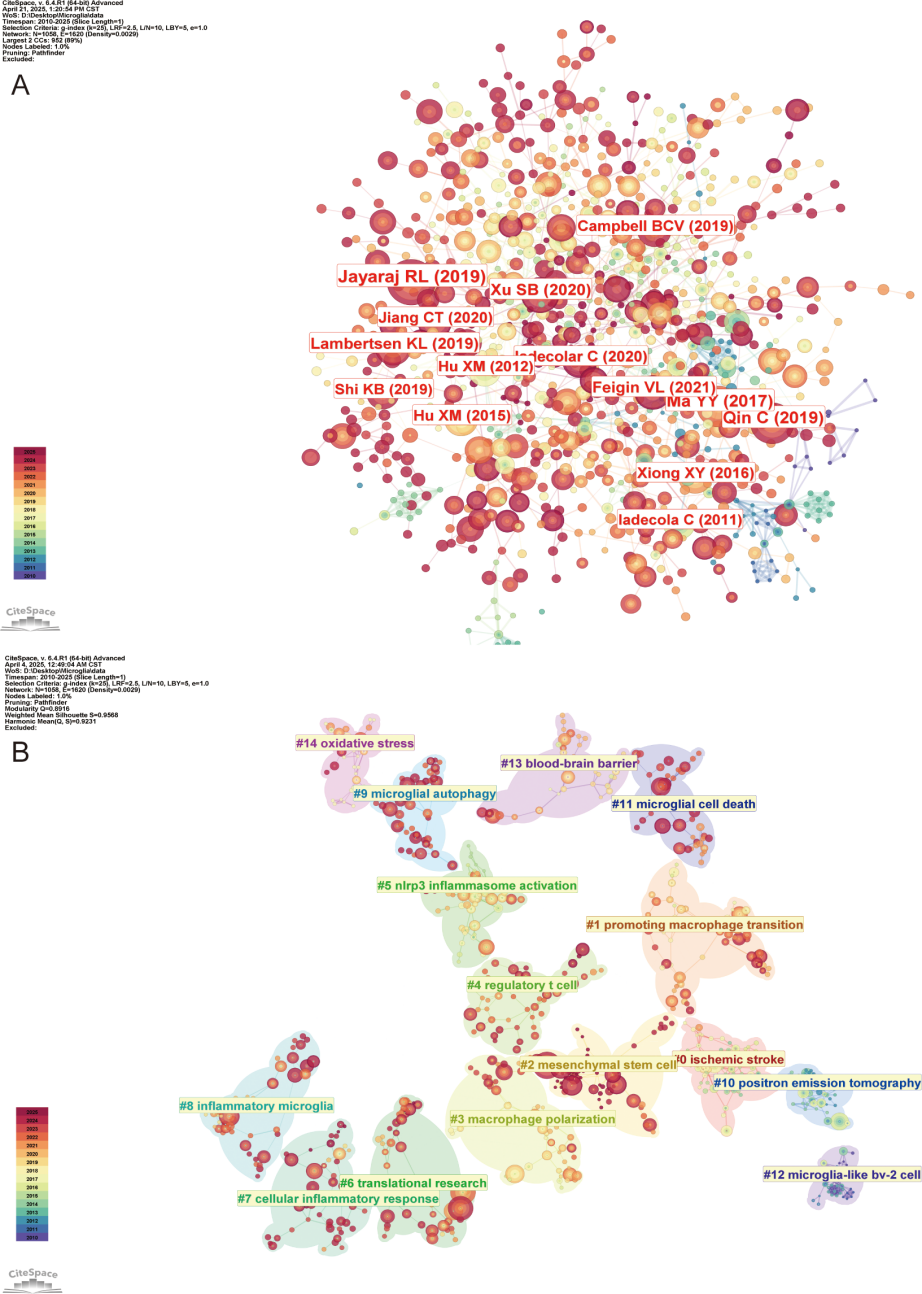
Visualization of co-cited references in the field of IS microglia research. **(A)** Visualization of the co-citation network of references. **(B)** Distribution of the top 15 major clusters of co-cited references.

The co-citation analysis of references provides valuable insight into the knowledge base of the research field and enables a more comprehensive exploration of its current state of development. **Figure 9A** illustrates the reference visualization map generated using CiteSpace, with a threshold set for references cited at least 60 times. **Table 6** lists the top 10 co-cited references by number of citations. Among them, Jayaraj RL (32), Xu SB (33), Lambertsen Kl (34), and Iadecola C (35) explored the critical role of microglia in neuroinflammatory responses following IS, emphasizing both their mediation of neurological damage and also their studies highlighted the importance of microglia in the recovery of neurological function. In contrast, the studies of Qin C (36), Ma YY (37), Jiang CT (10), and Hu XM (28) focused on the phenomenon of rapid activation of microglia following IS. These studies analyzed in detail the process of their polarization at different stages of IS through complex pathological mechanisms, ultimately leading to the formation of two distinct phenotypes that profoundly impact the progression and development of IS. Using CiteSpace for cluster analysis of the references, we identified a total of 28 clusters. **Figure 9B** presents the maps of the top 15 clusters.

**Table 6:** Top 10 most co-cited references in the field of microglia in IS.

Rank	Counts	Years	Co-cited references	First authors
1	154	2019	Neuroinflammation: friend and foe for ischemic stroke	Jayaraj RL (32)
2	128	2019	Dual Functions of Microglia in Ischemic Stroke	Qin C (36)
3	123	2017	The biphasic function of microglia in ischemic stroke	Ma YY (37)
4	93	2020	Glial Cells: Role of the Immune Response in Ischemic Stroke	Xu SB (33)
5	87	2019	Post-stroke inflammation—target or tool for therapy?	Lambertsen KL (34)
6	81	2021	Global, regional, and national burden of stroke and its risk factors, 1990–2019: a systematic analysis for the Global Burden of Disease Study 2019	Feigin VL (40)
7	77	2020	Modulators of microglia activation and polarization in ischemic stroke (Review)	Jiang CT (10)
8	77	2020	Immune responses to stroke: mechanisms, modulation, and therapeutic potential	Iadecolar C (35)
9	76	2019	Ischaemic stroke	Campbell BCV (41)
10	76	2015	Microglial and macrophage polarization—new prospects for brain repair	Hu XM (28)

These clusters suggest that IS (#0) is followed by triggering cerebral ischemia, which in turn leads to oxidative stress (#14) and disruption of BBB (#13). During this pathological process, microglia are activated (#8), releasing pro-inflammatory factors that activate NLRP3 inflammasomes (#5), thereby driving cellular inflammatory responses (#7). Neuroinflammatory processes may involve microglial autophagy (#9), which exerts both protective and damaging effects, as well as microglial cell death (#11) (apoptosis or pyroptosis), which can either exacerbate or alleviate inflammation. Additionally, research on macrophage polarization (#3), phenotypic transition (#1), and Tregs (#4) have revealed immunomodulatory targets in the field of IS microglia research. The MSCs (#2) and translational research (#6) focus on therapeutic strategies to convert the above mechanistic insights into clinical applications. Moreover, positron emission tomography (PET) imaging (#10) and BV-2 cell modeling (#12) are essential technological tools in the field of microglia research in IS. Therefore, it is evident that the mechanisms involving microglia in IS are complex and multifaceted.

**Figure 10:**
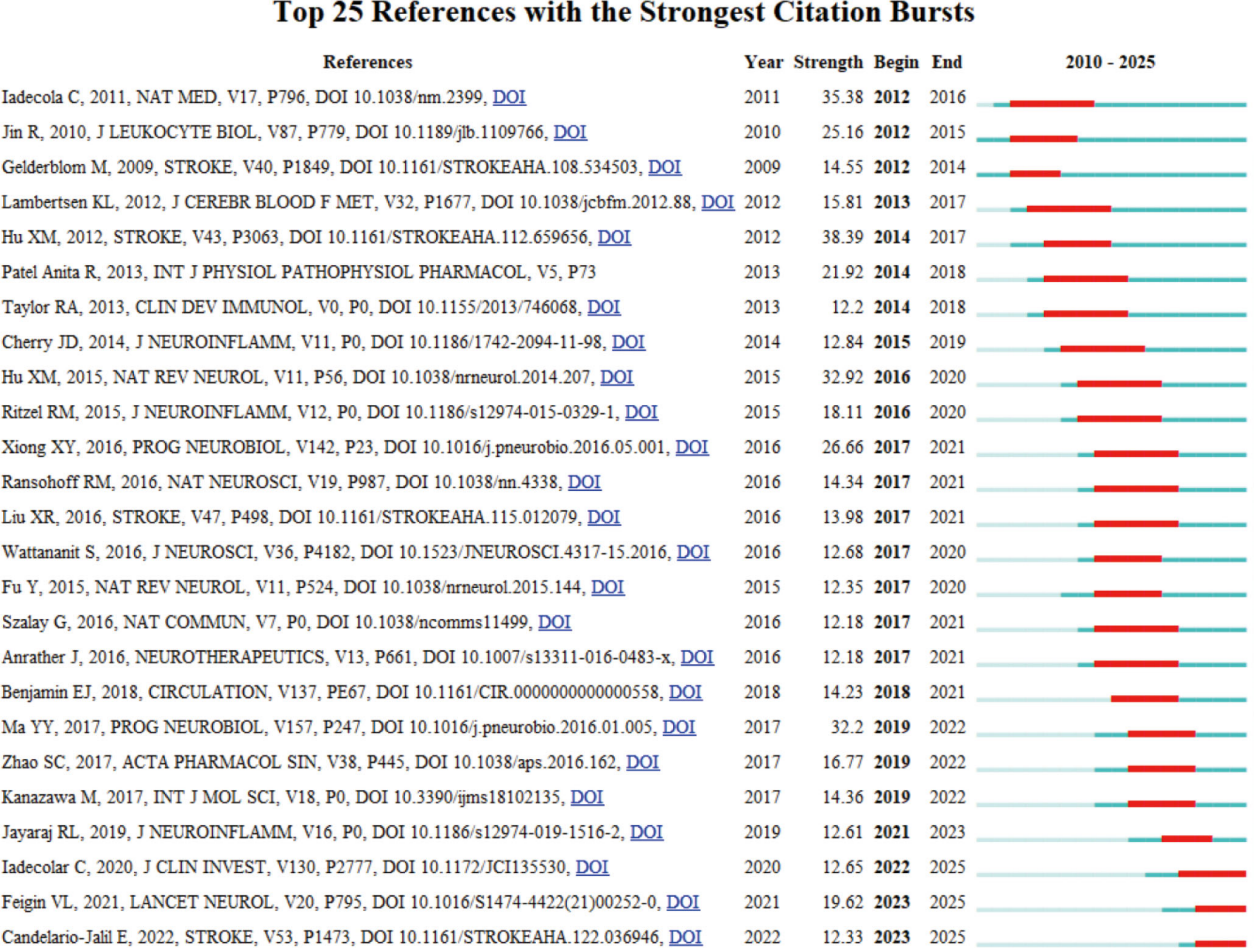
Top 25 references for burst strength in IS microglia research.

Citation Burst analysis of references helps identify influential literature over time. **Figure 10** reveals the Top 25 References with the Strongest Citation Bursts. Notably, the study by Hu XM (31) published in 2012 ranked first, with a citation burst intensity of 38.39. This study explored the mechanism underlying microglial polarization in IS. Following closely, a study by Iadecola C (38) in 2011 achieved a citation burst strength of 35.38, focusing on the key role of immunity and inflammation in stroke pathology and exploring the potential application of immunomodulation in IS. Another significant study by Hu XM (28), published in 2015, had a citation burst strength of 32.92 and mainly revealed the molecular mechanisms regulating microglia phenotypic changes. Furthermore, it is noteworthy that the study by Iadecola C (35) published in 2020, which explored the potential therapeutic value of targeting immunomodulatory factors in IS, continues to maintain a high burst intensity. Finally, the study by Candelario–Jalil E (39) published in 2022, focuses on imaging cellular or molecular markers of neuroinflammation, providing additional diagnostic insights. Collectively, these studies reflect emerging trends and growing research interest in the field.

## 4 Discussion

### 4.1 Summary of basic information

This study conducted a bibliometric analysis of 2,117 publications on microglia in IS between January 1, 2010, and March 15, 2025. The papers consisted of 1,774 articles and 343 reviews. The number of publications in the paper increased modestly until 2015, while the growth rate accelerated significantly following 2015. Overall, the number of papers published revealed a steady upward trend, indicating that microglia are still a hotspot of interest for researchers in the field of IS. Among the 60 countries analyzed, China topped the list with 1,240 papers published, followed by the United States (496 papers) and Germany (168 papers). China and the United States dominate the field in terms of influence. Besides, there is the closest cooperation between the United States and China, as well as Germany, and these countries have made significant contributions to the development of the field. A total of 1,993 institutions are involved in this study, of which nine of the top 10 institutions in terms of the number of publications are from China, and one is from the United States. Capital Medical University, Fudan University, and the University of Pittsburgh are the top three institutions with the highest impact. Increased collaboration with these institutions may drive further growth in this area. Furthermore, inter-institutional collaborations are primarily focused on 2021–2022 and beyond. In the analysis of 11,753 authors, Yun Xu from Nanjing University was identified as the most productive author with 42 publications, highlighting his research efficiency. He was followed by Dirk M. Hermann from Ruhr University Bochum (20 papers) and Jun Chen from the University of Pittsburgh (17 papers). Notably, Jun Chen from the University of Pittsburgh is recognized as the most influential scholar in the field. However, the collaborative network among core authors still needs to be further strengthened to foster more extensive exchanges and promote the rapid development of the field. Among the 48,684 co-cited authors, Xiaoming Hu from the University of Pittsburgh had the highest number of citations, with 603 fully reflecting the wide recognition of his research contributions. The 2,117 papers analyzed in this study were published across 465 journals, with the *Journal of Neuroinflammation*, *International Journal of Molecular Sciences*, and *CNS Neuroscience & Therapeutics* ranked among the top three in terms of the number of papers published, with a total of 202 papers published, demonstrating the vital contribution of these journals in the field. Notably, the *Journal of Neuroinflammation* is also the most influential in the field. In addition, *Stroke* stands out as the most cited journal, with 7,330 citations, demonstrating its authority in microglia research in IS.

### 4.2 Research hotspots and trends

**Figure 11:**
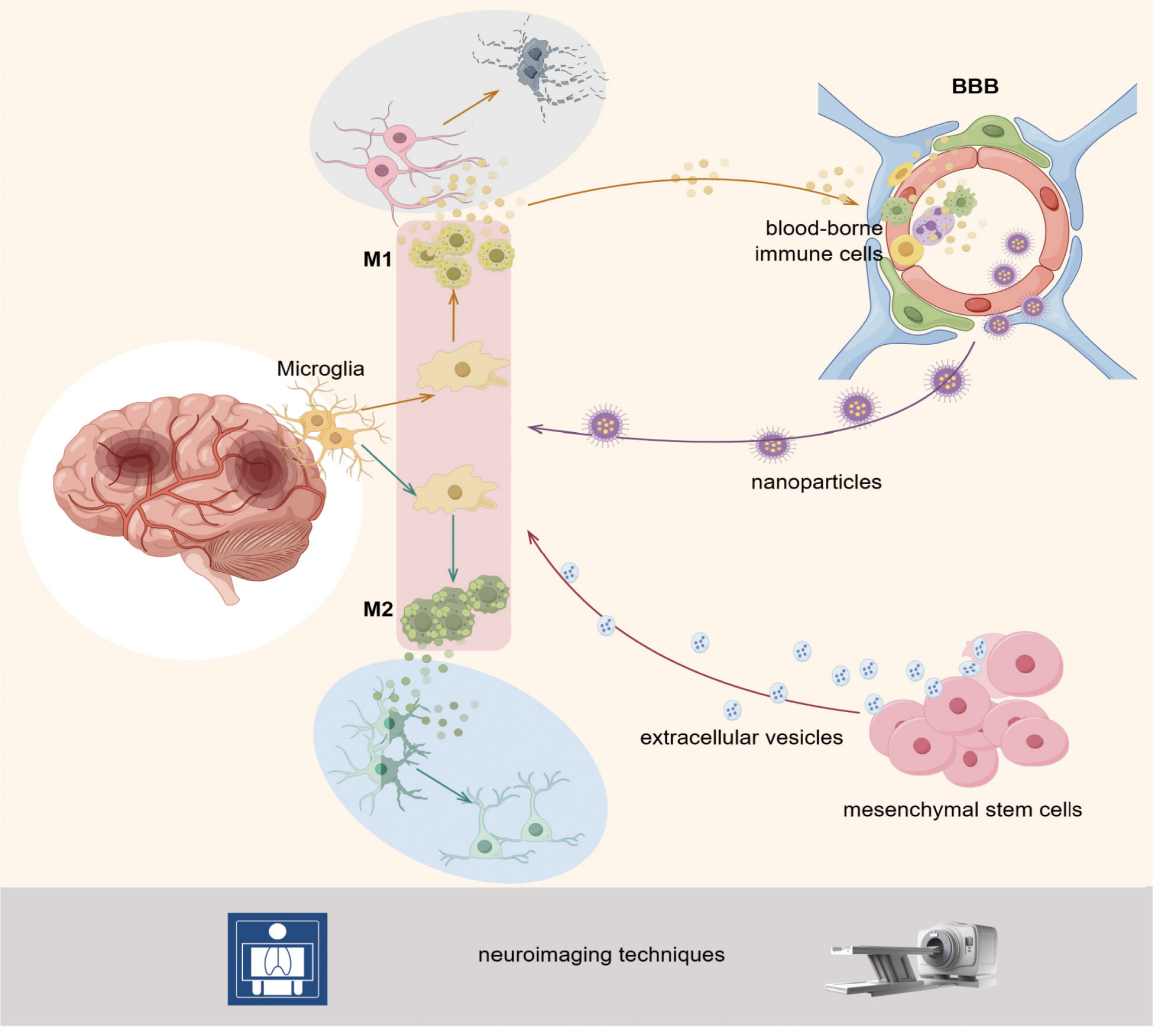
Following the onset of IS, microglia undergo rapid activation and polarization to either the M1 or M2 phenotype, contingent upon the pathological milieu. M1 microglia have been shown to release pro-inflammatory factors, which can lead to the injury and death of neurons. These microglia have also been observed to attract blood-derived cells to the blood-brain barrier, thereby exacerbating their damage. M2 microglia secrete anti-inflammatory and reparative factors, thereby promoting the recovery and regeneration of neurons. The therapeutic approach to this pathological process involves using mesenchymal stem cells, extracellular vesicles, and nanoparticle technology to target the state of the microglia. The aforementioned pathological changes and therapeutic processes can be monitored and evaluated with precision using PET, MRI, and other neuroimaging techniques. By Figdraw.

Through visual analysis of subject classification, keywords, and co-cited references, we identified research hotspots and trends in microglia in IS. The primary focus is on three areas: Mechanism, diagnosis, and treatment. **Figure 11** briefly illustrates the hotspots and trends in microglia research in IS.

#### 4.2.1 Triggering and regulation of neuroinflammation

In the pathological process of IS, inflammatory response and immunoregulatory mechanisms play a central role throughout the different disease development periods (42). The network of immune-inflammatory interactions within the CNS microenvironment exerts a complex impact on neurological function through its bidirectional regulatory properties. The direction of this action depends on several factors, including the type of immune cell subset activated, the effector molecule released, the location of the lesion, and the stage of the disease (15, 16). Activation and polarization of microglia, one of the earliest responders to ischemic injury, are central drivers of neuroinflammation (43). In response to pathological stimuli, resting microglia become activated, assume an amoeboid shape, and exhibit enhanced mobility, phagocytosis, and proliferation (44). Subsequently, microglia can be regulated by focal pathological signals to undergo phenotypic, developing either a classically activated phenotype (M1) or an alternatively activated phenotype (M2). The M1 type drives the neuroinflammatory cascade by upregulating pro-inflammatory mediators interleukin (IL)-1β, tumor necrosis factor (TNF)-α, and matrix metalloproteinase (MMP) expression (13). Recent studies have further revealed that the activation of the NLRP3 inflammasome in M1 microglia represents a central event in neuroinflammation. Activated NLRP3 inflammasomes trigger pyroptosis by cleaving gasdermin D protein and lead to the massive release of IL-1β and IL-18, thereby exacerbating the inflammatory response (45). In contrast, M2 polarization promotes tissue repair through the secretion of anti-inflammatory factors such as IL-10 and TGF-β, as well as tissue-repair mediators, and may contribute to BBB integrity by suppressing NLRP3 pathway activation (43, 46). It can be observed that the activation and polarization state of microglia, as well as the neuroinflammation or neurorepair process they trigger, have a central regulatory role in the occurrence, development, and prognosis of brain injury after IS. Consequently, it is a promising neuroprotective strategy to actively explore the interaction between microglia and neuroinflammation in IS development, to accurately regulate the polarization state of microglia using gene editing or targeted drugs, and then reshape the dynamic balance of inflammatory injury and anti-inflammatory repair.

#### 4.2.2 Interaction of the BBB with neuroinflammation

The BBB, as a key regulatory interface of the CNS, maintains the stability of the neural microenvironment through its selective substance transport mechanisms and plays a crucial role in meeting the metabolic demands of neurons and maintaining electrophysiological activity (47) The IS can disrupt the integrity of the BBB, as evidenced by the dissociation of tight junction (TJ) structures and enhanced function of the endothelial cell transport system. These changes ultimately lead to an abnormal increase in barrier permeability (48). BBB disruption triggers uncontrolled leakage of blood-borne cells, macromolecules, and fluids, which in turn leads to serious consequences such as severe cytotoxicity, vasogenic edema, and life-threatening HT (49). The release of inflammatory factors from activated microglia can further undermine the integrity of the BBB, creating a vicious cycle of peripheral immune cell infiltration and toxicant entry (50). The TNF-α secreted by M1 microglia was identified to be a major mediator of BBB destruction. The M1 microglia induce necrotic apoptosis in endothelial cells through the TNFR1-mediated pathway, leading to further BBB damage (14). In contrast, it has been found that the expression levels of serum pro-inflammatory factors and MMP-9 can be significantly reduced by inhibiting the activation of microglia and their polarization to the pro-inflammatory M1 phenotype while promoting their polarization to the M2 phenotype. This regulatory strategy not only effectively attenuates the structural damage to the BBB but also helps maintain BBB permeability homeostasis by upregulating the expression of TJ proteins (ZO-1, occludin), thereby providing essential support for neuroprotection (48, 50). Overall, the interaction between BBB and neuroinflammation primarily depends on the regulatory mechanisms mediated by microglia. Currently, targeting the activation and polarization regulation of microglia, as well as their interaction with endothelial cells, remains a significant research focus and trend in the field of IS therapy.

#### 4.2.3 Dynamic monitoring by neuroimaging techniques

Changes in microglia activation and polarization status, BBB permeability, and the distribution of inflammatory areas have become key indicators for defining the stage of IS disease and evaluating treatment efficacy (51). Currently, researchers can accurately capture the microstructural remodeling, functional network reorganization, and spatial distribution characteristics of the inflammatory microenvironment of brain tissues after IS using neuroimaging techniques (PET, magnetic resonance imaging [MRI]) (51, 52). The PET enables precise tracing of molecular probes related to neuroinflammation, among which the mitochondrial translocator protein (TSPO) is highly and specifically expressed in activated microglia, making it an important molecular target for characterizing the dynamic evolution of the neuroinflammatory response (53). The MRI is based on the blood oxygen level-dependent effect and visualizes brain function through the paramagnetic properties of endogenous deoxyhemoglobin. Although its detection principle technologically complements PET, MRI’s application range is constrained by its relatively low spatial resolution and high costs associated with equipment operation and maintenance (52). The new PET-MRI hybrid imaging system realizes the simultaneous acquisition of metabolic information and multi-parameter functional data by integrating the advantages of the two imaging technologies. This technology has been successfully applied in stroke research, which can not only reconstruct the spatial and temporal dynamics of immune cell infiltration in ischemic regions in three dimensions but also dynamically monitor the intensity of neuroinflammation, the process of microcirculatory remodeling, and the state of functional compensation, thus providing a multidimensional imaging basis for assessing therapeutic efficacy (54–56). Given that microglia play a dynamic regulatory role throughout the entire pathological process of IS, fully utilizing advanced neuroimaging techniques to dynamically monitor their activity and track their spatio-temporal distribution is expected to further elucidate the pathological mechanisms of IS and provide a crucial reference for individualized neuroimmunotherapy.

#### 4.2.4 Therapeutic strategies for MSCs and EVs

As an emerging therapeutic approach, MSC therapies are now widely studied and applied in treating IS (57). Available evidence suggests that most stem cells injected systemically are trapped in the lungs and do not effectively reach damaged tissues. Therefore, their therapeutic efficacy is, to a lesser extent, related to the direct differentiation of stem cells into damaged tissues (58). However, the therapeutic efficacy of MSCs may be primarily dependent on the “bystander effect,” for instance, the therapeutic efficacy exerted through the paracrine effects (59). The MSCs, through paracrine action, produce the MSCs’ secretome, which consists of a large number of cytokines (chemokines, neurotrophic factors) and EVs, which can directly contact the immune microenvironment following IS to modulate the state of microglia, inhibit neuroinflammation, and repair the BBB (60). Notably, EVs are key to the neuroprotective effects of MSCs. For instance, EV infusion in rats with focal brain injury significantly reduced the activation of glial cells and infiltrating immune cells in the brain tissue of the former compared with the untreated ones, which attenuated neuroinflammation following cerebral ischemia (61). It also significantly inhibited microglia M1-type polarization and activated the NRF2/NF-κB/NLRP3 pathway to induce neuroinflammation and oxidative stress, meanwhile increasing M2-type microglia infiltration to reduce neuroinflammation and promoting angiogenesis to exhibit neuroprotective functions (62–64). Moreover, EVs carry protein molecules, microRNA, and other bioactive molecules that can participate in intercellular communication. For instance, EVs ameliorate IS by transferring and releasing miR-23a-3p, which induces microglia inactivation and promotes their polarization towards the M2 type (65). Currently, many animal experiments have confirmed the potential of MSC-EVs in the treatment of IS. However, there are still many challenges to achieving clinical translation. Future clinical trials should focus on improving the safety and survival of MSCs and enhancing the targeting of EVs.

#### 4.2.4 Targeted applications of nanoparticle technology

Microglia participate in and regulate the progression of IS disease through complex response mechanisms, and this complexity makes effective regulation of their state challenging in many ways. Besides, the presence of the BBB further limits effective drug delivery (66). However, the interdisciplinary integration of nanomedicine has opened up innovative pathways to address these challenges (67). The nanoparticles (NPs)-based drug delivery systems have significantly enhanced intervention efficacy by targeting the delivery of microglia-specific regulatory molecules, breaking through the multiple limitations of traditional drug delivery modes regarding BBB penetration, cellular selectivity, and drug-controlled release ability (68). Notably, the design of NPs is becoming more refined to accommodate better complex regulatory mechanisms, which has driven the application of different types of NPs in microglia-targeted drug delivery. For instance, in a mouse model of MCAO, APTS, a neutrophil trapping nanoplatform, significantly enhanced the delivery efficiency of ischemic tissues to A151, a telomerase repeat sequence drug, by 4-fold. The A151, through the platform, with the aid of the reactive oxygen scavenging-mediated inhibition of the guanosine histone three pathway, decreased the efficiency of neutrophil extracellular trapping networks (NETs) formation by 2.2-fold and contributed to the polarization of microglia towards an anti-inflammatory M2 phenotype (69). Meanwhile, plant-derived exosome-like NPs have indicated therapeutic potential. Panax ginseng-derived exosome-like NPs crossed the BBB without modification and directly entered the brain, significantly attenuating cerebral ischemia/reperfusion injury by shifting microglia phenotypes from M1 to M2 phenotype. This process may be associated with activating the PI3k/Akt signaling pathway through lipid components (70). Furthermore, a novel lipid NPs platform targeting M2 microglia constructed a protective feedback loop promoting microglia polarization by delivering messenger ribonucleic acid encoding IL-10. This process enhanced microglia M2 polarization in the MCAO model, accelerated neuroinflammatory regression, restored BBB function, and inhibited neuronal apoptosis. The therapy also prolonged the therapeutic time window of IS to 72 h (71). By precisely regulating the state of microglia, these technologies provide efficient, controllable, and easy-to-produce novel means of immunotherapy for cerebral ischemia and related diseases, demonstrating broad application prospects.

### 4.3 Challenges and current status in clinical translation

This study reveals that MSC-EVs show significant preclinical potential in regulating microglial polarization, alleviating neuroinflammation, and reducing oxidative stress. Therefore, MSC-EVs are considered one of the most promising strategies for the treatment of ischemic stroke. However, several key challenges remain for their clinical translation.

Currently, there are various methods for isolating extracellular vesicles (EVs), but standardization of production remains a major bottleneck (72). Variations in the content and proportions of functional components (such as miR-23a-3p or IL-10) across different batches of EVs directly affect the consistency and reproducibility of their effects on microglial polarization regulation. Additionally, regulatory frameworks are still underdeveloped (73). Given the complex composition of EVs, it is crucial to identify their key active components and mechanisms of action and establish corresponding quality control standards to meet drug evaluation requirements.

Notably, improving the efficiency of brain-targeted delivery has become an urgent priority, influenced by multiple factors. This is not only critical for enhancing therapeutic efficacy but also central to advancing clinical translation (74). To address this challenge, nanotechnology can be used to engineer the surface of EVs, such as by conjugating them with microglia-specific ligands to enhance their ability to cross the blood-brain barrier and accumulate in ischemic regions, thereby improving therapeutic outcomes and reducing systemic exposure risks (74). Simultaneously, modifying the administration route during nanotechnological engineering can be beneficial. For instance, studies have shown that incorporating neurotrophic peptides like BDNF into MSC-EVs using non-destructive methods and administering them via intranasal delivery significantly enhances post-stroke neuroregeneration. These EVs maintain good structural integrity and functional activity in large-scale clinical preparations and demonstrate neurogenesis-promoting and neuroprotective effects in both *in vitro* and *in vivo* experiments (75). Furthermore, research indicates that EVs derived from MSCs cultured in 3D using gelatin methacryloyl hydrogels (a biomimetic nanofiber microenvironment) exhibit superior performance in ischemic targeting, microglial uptake, and functional activities such as pro-angiogenesis and anti-inflammation (63).

Notably, a Phase I/IIa clinical trial (NCT07143786) is currently evaluating the safety and preliminary efficacy of intravenous administration of human induced neural stem cell-derived exosomes for the treatment of acute ischemic stroke. This marks a significant step forward in the clinical application of EV therapy. In terms of efficacy assessment, the development of reliable biomarkers is crucial. Currently, an ongoing clinical trial since 2020 (NCT04412187) employs TSPO-PET imaging technology to dynamically monitor the phenotypic switching of microglia and systematically assess the evolution of neuroinflammation post-stroke. This study provides an important evaluation model for the clinical translation of EV therapies.

### 4.4 Limitations

This study has several limitations. During the literature retrieval process, data collection was conducted exclusively using the WoSCC to ensure high-quality data. However, this approach did not include other databases such as Scopus and PubMed, which could result in a lack of breadth in the data analysis. Moreover, the CiteSpace analysis has certain shortcomings, such as flaws in author identification (the inability to differentiate between specific roles of authors and the problem of homonymity). The lack of standardized clustering parameters may also introduce data bias.

In the future, we will closely monitor the research dynamics in this field. If necessary, we will implement a multi-database comprehensive search strategy, conducting comparative and complementary analyses to validate and optimize our existing research conclusions. Additionally, we recommend actively utilizing artificial intelligence (AI) technologies to establish standardized systems for software parameters and develop high-precision author disambiguation systems. These efforts aim to enhance the reliability, depth, and reproducibility of bibliometric analyses.

## 5 Conclusion and prospects

This study employed bibliometric methods to conduct a systematic analysis of the literature related to microglia in IS. The analysis identified three core research directions in this field: neuroinflammation mechanisms, dynamic imaging monitoring, and targeted therapeutic strategies. Microglia, as a central component of immune regulation in the central nervous system, play a crucial role in neuroinflammatory responses and BBB disruption through their M1/M2 polarization dynamics and the NLRP3/GSDMD-mediated pyroptosis pathway. In terms of therapeutic strategies, MSC-EVs have shown significant neuroprotective and anti-inflammatory effects by modulating microglial polarization states. This makes MSC-EVs one of the most promising translational therapies currently available. Additionally, nanoparticle drug delivery systems can overcome BBB limitations, significantly extend the therapeutic time window, and enhance intervention efficiency. Notably, based on nanoparticle delivery technology, future treatments may achieve precise delivery of NLRP3 inflammasome inhibitors (such as MCC950) or GSDMD neutralizing antibodies to ischemic brain regions. This would enable precise modulation of microglial function, providing new directions for the treatment of IS.

Despite the promising potential of MSC-EVs, their clinical translation still faces several significant challenges, including production standardization, enhancing brain-targeted delivery efficiency, and establishing regulatory frameworks. Future research should focus on several key areas. 1) Establishing stable and scalable production processes and quality control systems for EVs to ensure their safety and efficacy in clinical applications. 2)Developing engineering modification strategies to enhance targeting to lesioned microglia, such as improving targeting to ischemic regions through surface functionalization. 3)Conducting in-depth studies on the active components and mechanisms of action to promote the development of regulatory science pathways, providing a solid theoretical foundation for clinical use. Additionally, developing dynamic evaluation systems based on imaging technologies such as TSPO-PET for real-time monitoring and therapeutic efficacy prediction during treatment is critical for advancing personalized therapy.

Looking ahead, through interdisciplinary integration and technological innovation, diagnostic and therapeutic strategies targeting microglia are expected to provide a comprehensive solution from mechanism elucidation, imaging assessment, to precise intervention for ischemic stroke. With continuous breakthroughs in EV engineering technology, nanomedicine, and multimodal imaging techniques, treatment strategies aimed at modulating microglial polarization to improve stroke outcomes are likely to gradually move towards clinical application.

## Data Availability

The original contributions presented in the study are included in the article/**Supplementary Material**. Further inquiries can be directed to the corresponding author.
