# Supplementary material for: Global research hotspots and trends in microglia in ischemic stroke
Source: Front Immunol. 2025 Oct 31;16:1622499. doi: 10.3389/fimmu.2025.1622499 (PMC12635585; doi:10.3389/fimmu.2025.1622499)
Supplement: Supplementary file 1 [file Table1.docx]

**1. Scopus search formula**

TITLE-ABS-KEY ( "microglia" OR "microglias" OR "microglial cell" OR "cell, microglial" OR "microglial cells" ) AND TITLE-ABS-KEY ( "ischemic stroke" OR "ischemic strokes" OR "stroke, ischemic" OR "acute ischemic stroke" OR "acute ischemic strokes" OR "cryptogenic ischemic stroke" OR "cryptogenic stroke" OR "cryptogenic embolism stroke" )

1. **Scopus data screening process**

**Topic:** Microglia in ischemic stroke

**Timespan:** 2010-01-01 to 2025-03-15 (publication date)

**Search Date:** 2025-09-17

**2801** results identified

**2238** results identified

**Exclude:**
Not relevant to the topic(n=258)

**Exclude:**
Not English (n=53)

To ensure consistency in retrieval criteria, papers published between 2025-03-16, and 2025-09-17 were manually excluded (n=197).

**Exclude：**
Editorial (n=32)
Book chapter (n=27）

Short survey (n=13)
Retracted (n=12)
Note (n=9)
Erratum (n=8)
Letter (n=3)

Conference paper (n=3)

Conference review (n=1)

**2854** results from Scopus

**2496** results identified
